# Multitrait genome-wide association best linear unbiased prediction of genetic values

**DOI:** 10.1186/s12711-025-00964-4

**Published:** 2025-03-21

**Authors:** Theo Meuwissen, Vinzent Boerner

**Affiliations:** 1 Faculty of Life Sciences, Norwegian University of Life Sciences, 1432 Ås, Norway; 2 GHPC Consulting and Service PTY, LTD, Armidale, Australia

## Abstract

**Background:**

The GWABLUP (Genome-Wide Association based Best Linear Unbiased Prediction) approach used GWA analysis results to differentially weigh the SNPs in genomic prediction, and was found to improve the reliabilities of genomic predictions. However, the proposed multitrait GWABLUP method assumed that the SNP weights were the same across the traits. Here we extended and validated the multitrait GWABLUP method towards using trait specific SNP weights.

**Results:**

In a 3-trait dairy data set, multitrait GWAS estimates of SNP effects and their standard errors were translated into trait specific likelihood ratios for the SNPs having trait effects, and posterior probabilities using the GWABLUP approach. This produced trait specific prior (co)variance matrices for each SNP, which were applied in a SNP-BLUP model for genomic predictions, implemented in the APEX linear model suite. In a validation population, the trait specific SNP weights resulted in more reliable predictions for all three traits. Especially, for somatic cell count, which was hardly related to the other traits, the use of the same weights across all traits was harming genomic predictions. The use of trait specific SNP weights overcame this problem.

**Conclusions:**

In multitrait GWABLUP analyses of ~ 30,000 reference population cows, trait specific SNP weights resulted in up to 13% more reliable genomic predictions than unweighted SNP-BLUP, and improved genomic predictions for all three studied traits.

## Background

GBLUP (Genomic Best Linear Unbiased Prediction) and SNP (Single Nucleotide Polymorphism)-BLUP [1] are currently the most commonly used genomic prediction methods. Especially, GBLUP is relatively simple to use and aligns closely with earlier breeding value evaluation methods. Moreover, these methods are equivalent and yield identical prediction accuracies [2]. Hence, we can choose the method that best meets our needs. In addition, they can be extended to single step predictions, which combines information from genotyped and non-genotyped individuals [3].

A limitation of GBLUP and SNP-BLUP is their assumption that all SNPs contribute equally to the total genetic variance. Bayesian variable selection methods (e.g. BayesA, BayesB, BayesC, and BayesR [1, 4, 5]) allocate more variance or weight to the most important SNPs but are complex and computationally demanding using Monte Carlo Markov Chain (MCMC) sampling techniques. Recently, GWABLUP was proposed which uses deterministic weights based on GWAS (Genome Wide Association Study) results [6]. Since the SNP weights are (pre)determined by the GWAS signals, GWABLUP is based either on a weighted SNP-BLUP or on a weighted G-matrix in GBLUP, which may both be extended to single step methods (ssGWABLUP).

Also a multitrait extension of GWABLUP was proposed assuming that SNP weights are equal across the traits [6]. The latter assumed that all traits are affected by the same QTL (Quantitative Trait Loci). But generally, different traits will be affected by at least partly different QTL, and the use of the same SNP weights across the traits is suboptimal. Our aim is here to extend the multitrait-GWABLUP method to using SNP weights that are trait specific and to compare the results to using equal weights across the traits. The methods are compared in the same dairy cattle data set as [6], and using the APEX linear models suite (www.ghpc.ai), which implements multitrait SNP-BLUP with different (co)variance matrices per SNP and thus allows for different weights per SNP and per trait.

## Methods

### Data

The 3-trait dairy data set of [6] included the yield deviations (YD) of milk and protein yield and somatic cell count (SCC) and their reliability on 32,201 Norwegian Red cows, and was kindly provided by Geno SA (www.geno.no). Estimates of heritabilities, genetic and environmental correlations of the traits are depicted in Table 1. In [6], a canonically transformation of the 3 traits was performed [7] which resulted into 3 genetically and environmentally independent canonical traits with standardised environmental variances of 1 and genetic variances of 0.16, 0.29 and 1.44. The data also included imputed HD genotypes on *N*_*snps*_ = 617,739 SNPs for all 32,201 cows (see [6] for details).

**Table 1:** Heritabilities (diagonals), genetic correlations (below diagonals), and residual correlations (above diagonals) of the dairy traits

	Milk	Prot	SCC
Milk	0.26	0.96	− 0.17
Protein	0.85	0.20	0.16
SCC	0.10	0.10	0.16

### SNP weights

Uniform SNP-weights across the traits were obtained from the combined log-likelihood ratios of the GWAS of the 3 canonical traits as described in [6]. The GWAS of the three canonical traits resulted also in estimates of their SNP effects and standard errors for each of the canonical traits. To obtain trait specific SNP weights, these canonical trait SNP effects were back-transformed to original trait SNP effects together with their standard errors. Following [6], GWABLUP loglikelihood ratios per SNP and trait were calculated as:

1 $${LR}_{tj}=0.5\frac{\widehat{{b}_{tj}^{2}}}{s{e}_{tj}^{2}},$$

where ${LR}_{tj}$ is the log-likelihood ratio of SNP *j* for trait *t*, $\widehat{{b}_{tj}}$ is the GWAS estimate of the effect of SNP *j* for trait *t*, and $s{e}_{tj}$ is the standard error of $\widehat{{b}_{tj}}$. The model averaging of MCMC Bayesian genomic prediction schemes is to some extend mimicked by calculating the moving average of the likelihood ratios, i.e., by averaging the likelihood ratios of the current SNP and two adjacent SNPs to the left and two adjacent SNPs to the right, resulting in ${\overline{LR} }_{tj}$. Following [6], posterior probabilities that the SNPs have non-zero effects were calculated for trait *t* and SNP *j* as:

$$P{P}_{tj}=\pi {e}^{{\overline{LR} }_{tj}}/[\pi {e}^{{\overline{LR} }_{tj}}+(1-\pi )],$$

which serve as trait specific weights for the SNPs, where the prior probability of non-zero SNP effects is $\pi =0.001$.

### Multitrait SNP-BLUP analyses

The model for the multitrait SNP-BLUP is:

2 $$\mathbf{y}=\mathbf{X}{\varvec{\upmu}}+\mathbf{Z}\mathbf{b}+\mathbf{e},$$

where $\mathbf{y}$ is a vector of yield deviations for the 3 traits (ordered by traits within cows); ${\mathbf{X}}=\mathbf{1}_{{{\varvec{n}}}_{{\varvec{a}}}}\otimes {\mathbf{I}}_{3}$ is a design matrix linking the records to their trait means (${n}_{a}$ is the number of animals); ${\varvec{\upmu}}$ is a vector of estimates of trait means for the 3 traits; $\mathbf{Z}=\mathbf{M}\otimes {\mathbf{I}}_{3}$ with **M** being a (${n}_{a}$ ×* N*_*snps*_) matrix of centred allele counts of the cows; $\mathbf{b}$ is a vector of (3* N_*snps*_) multitrait SNP effects (ordered by traits within SNPs). The prior distribution of the residuals was $\mathbf{e}\sim N(\mathbf{0},\left(\mathbf{W}\otimes \mathbf{R}\right))$, where **R** is the residual (co)variance matrix of the traits (Table 1) and $\mathbf{W}$ is a (${n}_{a}$ × ${n}_{a}$) diagonal matrix with the inverses of the weights of the yield deviations on the diagonal.

For the analysis of model (2), the multitrait SNP-BLUP method of [8–10] is used, where in our data all animals are genotyped and pedigree relationships are not used and thus set to an identity matrix. The (co)variance matrix of the breeding values and SNP effects (traits within cows and SNPs) is modelled by:

$$Var\left(\left[\begin{array}{c}\mathbf{u}\\ \mathbf{b}\end{array}\right]\right)=\left[\begin{array}{cc}\mathbf{Z}{\varvec{\uptheta}}\mathbf{Z}^{\prime}+\varepsilon {\mathbf{I}}_{{{\varvec{n}}}_{{\varvec{a}}}}& \mathbf{Z}{\varvec{\uptheta}}\\ {\varvec{\uptheta}}\mathbf{Z}^{\prime}& {\varvec{\uptheta}}\end{array}\right],$$

where $\mathbf{u}=\mathbf{Z}\mathbf{b}$ is a vector of multitrait breeding values; $\varepsilon$ is a small number ($\varepsilon$ = 0.01 was used here) added to regularize the matrix and making $$Var\left(\left[\begin{array}{c}\mathbf{u}\\ \mathbf{b}\end{array}\right]\right)$$ non-singular ($\varepsilon {\mathbf{I}}_{{{\varvec{n}}}_{{\varvec{a}}}}$ may be replaced by $\upvarepsilon {\mathbf{\rm A}} \mathbf{\otimes} {\mathbf{G}}$, where **G** is the genetic (co)variance matrix of the traits, to give some weight to the pedigree relationship matrix **A**, but this was not done here); and ${\varvec{\Theta}}$ is a (3*N*_*snps*_ × 3*N*_*snps*_) block-diagonal matrix of (3 × 3)-blocks:

$${\varvec{\Theta}}=\sum_{\oplus }{{\mathbf{V}}_{\mathbf{S}\mathbf{N}\mathbf{P}}}_{\mathbf{j}},$$

where $\oplus$ denotes the direct sum across all SNPs; ${{\mathbf{V}}_{\mathbf{S}\mathbf{N}\mathbf{P}}}_{\mathbf{j}}$ is the (3 × 3) SNP specific (co)variance matrix across the 3 traits, i.e. the SNP effects are a priori assumed unrelated with prior distributions ${\mathbf{b}}_{\mathbf{j}}\sim N(\mathbf{0},{{\mathbf{V}}_{\mathbf{S}\mathbf{N}\mathbf{P}}}_{\mathbf{j}})$, where ${\mathbf{b}}_{\mathbf{j}}$ is a (3 × 1) vector of the SNP effects of SNP *j*.

In case of regular multitrait unweighted SNP-BLUP (SNP_unw_-BLUP), i.e. unweighted SNP effects, the ${{\mathbf{V}}_{\mathbf{S}\mathbf{N}\mathbf{P}}}_{\mathbf{j}}$ matrices are the same for all SNPs *j*, i.e. ${{\mathbf{V}}_{\mathbf{S}\mathbf{N}\mathbf{P}}}_{\mathbf{j}}={\mathbf{V}}_{\mathbf{S}\mathbf{N}\mathbf{P}}$**,** and we have:

$${\mathbf{V}}_{\mathbf{S}\mathbf{N}\mathbf{P}}=\mathbf{G}/\sum_{j=1}^{{N}_{snps}}{2p}_{j}\left(1-{p}_{j}\right),$$

where **G** is the genetic (co)variance matrix of the traits (see Table 1) and ${p}_{j}$ is the allele-frequency of SNP *j*.

In case of equal weights across the traits (SNP_eqw_-BLUP), i.e. the SNP weights are used but weights are equal for all 3 traits as in the multitrait GWABLUP model in [6], the SNP specific (co)variance matrix is:

$${{\mathbf{V}}_{\mathbf{S}\mathbf{N}\mathbf{P}}}_{\mathbf{j}}={\mathbf{V}}_{\mathbf{S}\mathbf{N}\mathbf{P}}\frac{P{P}_{j}}{mean\left(P{P}_{j}\right)},$$

where $P{P}_{j}$ is the multitrait posterior probability of SNP *j* accumulated over the 3 traits, which is based on the sum of the loglikelihood ratios across the 3 canonical traits [6], and $mean(P{P}_{j})$ is the average of the $P{P}_{j}$’s.

In case of SNP and trait specific weights (SNP_tsw_-BLUP), the variances of the ${\mathbf{V}}_{\mathbf{S}\mathbf{N}\mathbf{P}}$ matrix are adjusted for the weights but not the correlations implied by ${\mathbf{V}}_{\mathbf{S}\mathbf{N}\mathbf{P}}$. This is achieved by:

3 $${{\mathbf{V}}_{\mathbf{S}\mathbf{N}\mathbf{P}}}_{\mathbf{j}}={{\mathbf{S}}_{\mathbf{j}}\mathbf{V}}_{\mathbf{S}\mathbf{N}\mathbf{P}}{\mathbf{S}}_{\mathbf{j}},$$

where ${\mathbf{S}}_{\mathbf{j}}$ is a 3 × 3 diagonal matrix with the *t*-th element:

$${{S}_{j}}_{tt}=\sqrt{\frac{P{P}_{tj}}{mea{n}_{j}(P{P}_{tj})}},$$

where $mea{n}_{j}(P{P}_{tj})$ denotes the average of the $P{P}_{tj}$’s over the SNPs *j* for trait *t*.

After constructing ${\varvec{\Theta}}$, the $$Var\left(\left[\begin{array}{c}\mathbf{u}\\ \mathbf{b}\end{array}\right]\right)$$ covariance structure, and its inverse, Henderson’s [11] mixed model equations (MME) were setup as [8]:

$$\left[\begin{array}{ccc}{\mathbf{X}^{\prime}}\left({\mathbf{W}}^{-1}\otimes {\mathbf{R}}^{-1}\right)\mathbf{X}&\mathbf{X}^{\prime}\left({\mathbf{W}}^{-1}\otimes {\mathbf{R}}^{-1}\right)& \mathbf{0}\\ \left({\mathbf{W}}^{-1}\otimes {\mathbf{R}}^{-1}\right)\mathbf{X}&\left({\mathbf{W}}^{-1}\otimes {\mathbf{R}}^{-1}\right)+{\mathbf{I}}_{{{\varvec{n}}}_{{\varvec{a}}}}/\varepsilon &-\mathbf{Z}/\varepsilon \\ \mathbf{0}& -{\mathbf{Z}^{\prime}}/\varepsilon &{\mathbf{Z}}^{\prime}\mathbf{Z}/\varepsilon +{{\varvec{\Theta}}}^{-1}\end{array}\right]\left[\begin{array}{c}\widehat{{\varvec{\upmu}}}\\ \widehat{\mathbf{u}}\\ \widehat{\mathbf{b}}\end{array}\right]=\left[\begin{array}{c}\mathbf{X}\left({\mathbf{W}}^{-1}\otimes {\mathbf{R}}^{-1}\right)\mathbf{y}\\ \left({\mathbf{W}}^{-1}\otimes {\mathbf{R}}^{-1}\right)\mathbf{y}\\ \mathbf{0}\end{array}\right],$$

An efficient iterative double-preconditioned conjugate gradient algorithm [12] was used to solve these equations as implemented in the APEX linear models suite (www.ghpc.ai; [9]), where the block diagonal preconditioner consisted of inverted diagonal blocks of the MME matrix of each level of the effects. This analysis of the SNP_unw_-BLUP, SNP_eqw_-BLUP and SNP_tsw_-BLUP models yielded multitrait estimates of SNP effects and of the animal’s breeding values.

### Validation cows

The records of the youngest cows born in 2018 (1988 cows) were used for validation, and their YDs were masked from the above data analyses. The remaining 30,213 cows were used for the training of the models, i.e. they were used for the GWAS analyses and the estimation of SNP effects. These SNP effects were used to obtain multitrait breeding value estimates of the validation cows as:

$${\widehat{\mathbf{g}}}_{\mathbf{v}}= {\mathbf{Z}}_{\mathbf{v}}\widehat{\mathbf{b}},$$

where ${\mathbf{Z}}_{\mathbf{v}}$ are the centered genotypes of the validation cows. The squared correlation between the predicted ${\widehat{\mathbf{g}}}_{\mathbf{v}}$ of the 1988 validation cows and their YDs (${\mathbf{y}}_{\mathbf{v}}$) were used as an indicator of the reliabilities of the ${\widehat{\mathbf{g}}}_{\mathbf{v}}$. These reliabilities were investigated for statistically significant differences (P < 0.05) by bootstrapping [13], which tests for significant differences between $cor\left({\mathbf{y}}_{\mathbf{v}},{\widehat{\mathbf{g}}}_{\mathbf{v}}^{\mathbf{k}}\right)$ and $cor\left({\mathbf{y}}_{\mathbf{v}},{\widehat{\mathbf{g}}}_{\mathbf{v}}^{\text{l}}\right)$, where superscripts denote prediction methods *k* and *l*. Bootstrap samples are obtained by sampling with replacement validation individuals, i.e. their ${\mathbf{y}}_{\mathbf{v}},{\widehat{\mathbf{g}}}_{\mathbf{v}}^{\mathbf{k}}$, and ${\widehat{\mathbf{g}}}_{\mathbf{v}}^{\mathbf{l}}$ – values. For each of 10,000 bootstrap data sets, $cor\left({\mathbf{y}}_{\mathbf{v}},{\widehat{\mathbf{g}}}_{\mathbf{v}}^{\mathbf{k}}\right)$ and $cor\left({\mathbf{y}}_{\mathbf{v}},{\widehat{\mathbf{g}}}_{\mathbf{v}}^{\text{l}}\right)$ is calculated, and its scored in how many of the data sets $cor\left({\mathbf{y}}_{\mathbf{v}},{\widehat{\mathbf{g}}}_{\mathbf{v}}^{\mathbf{k}}\right)$>$cor\left({\mathbf{y}}_{\mathbf{v}},{\widehat{\mathbf{g}}}_{\mathbf{v}}^{\text{l}}\right)$, where the comparison $cor\left({\mathbf{y}}_{\mathbf{v}},{\widehat{\mathbf{g}}}_{\mathbf{v}}^{\mathbf{k}}\right)$>$cor\left({\mathbf{y}}_{\mathbf{v}},{\widehat{\mathbf{g}}}_{\mathbf{v}}^{\text{l}}\right)$ is statistically significant if this is the case for > 97.5% of the bootstrap data sets.

## Results and discussion

The analyses of the SNP_unw_-BLUP, SNP_eqw_-BLUP and SNP_tsw_-BLUP models converged in 451, 2446, and 5211 iterations, respectively, using the Euclidean norm of the residuals of the equations relative to that of the right-hand-side < 10^–9^ as convergence criterion. Hence, the use of SNP weights and especially trait specific SNP weights slowed down the convergence of the models substantially.

**Fig. 1:**
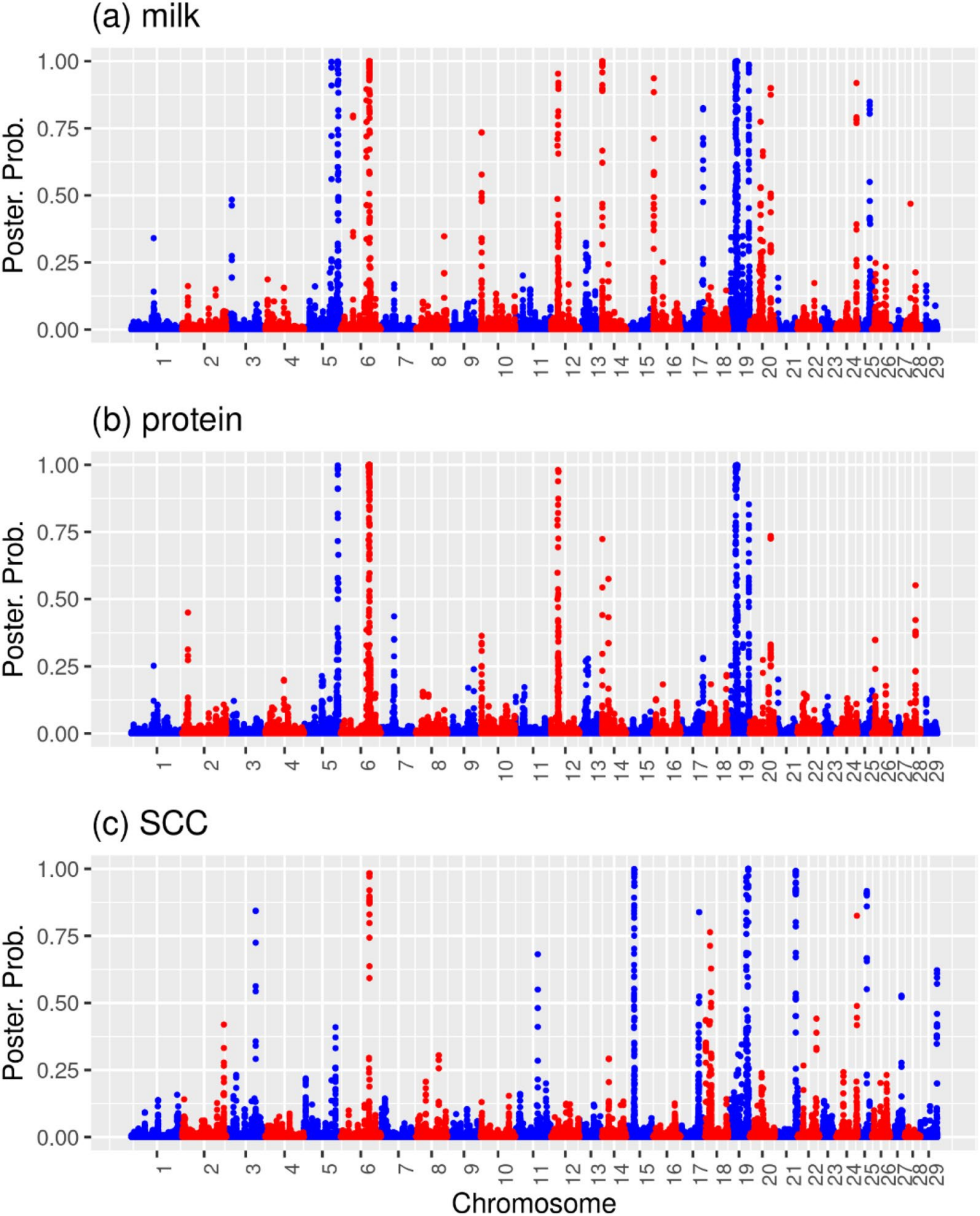
Manhattan plots of posterior probabilities of major effect SNPs. For milk (**a**), protein (**b**) yields and somatic cell count (**c**)

Figure 1 shows the posterior probabilities of non-zero effect SNPs, $P{P}_{tj}$, for each of the traits, which are proportional to the trait specific SNP weights used in the SNP_tsw_-BLUP analysis. Milk yield has $P{P}_{mlkj}$>0.9 SNPs on chromosomes 5, 6, 12, 14, 16, 19, and 24. Protein yield has $P{P}_{protj}$>0.9 SNPs on chromosomes 5, 6, 12, and 19. SCC has $P{P}_{SCCj}$>0.9 SNPs on chromosomes 6, 15, 19, 21, and 25. Figure 1 shows many posterior probability peaks, especially for milk yield, and the peaks for milk and protein yield show substantial overlaps, which is much less the case for the yield traits and SCC.

Table 2 shows the reliabilities of the multitrait genomic predictions measured as the squared correlations between GEBV and YDs for milk- and protein yields and SCC of the 1988 validation cows. The YDs have reliabilities of 0.409, 0.326, and 0.246, respectively [6], i.e., expressed relative to the YD reliabilities the reliabilities of SNP_unw_-BLUP are 0.49 (= 0.199/0.409), 0.54, and 0.68, respectively. For milk- and protein yields, genomic prediction reliabilities significantly increased by 11–13% using SNP weights, i.e. using the SNP_teqw_-BLUP and SNP_tsw_-BLUP models. Using trait specific weights (SNP_tsw_-BLUP) resulted in somewhat higher reliabilities than the SNP_eqw_-BLUP, although these differences were not statistically significant. SNP_tsw_-BLUP obtained the highest reliability for all three traits, albeit the improvement for SCC was minor and not statistically significant.

**Table 2:** Reliabilities of genomic predictions measured as the squared correlations between GEBVs and yield deviations of 1988 validation cows

	${\varvec{c}}{\varvec{o}}{\varvec{r}}{\left({\mathbf{y}}_{{\varvec{v}}},{\widehat{\mathbf{g}}}_{{\varvec{v}}}\right)}^{2}$^*,**^		
	Milk	Protein	SCC
SNP_unw_-BLUP^***^	0.199^a^	0.178^a^	0.168^a^
SNP_teqw_-BLUP^***^	0.223^b^	0.197^b^	0.160^a^
SNP_tsw_-BLUP^***^	0.226^b^	0.201^b^	0.169^a^

^*^Standard errors of $cor{\left({\mathbf{y}}_{{\varvec{v}}},{\widehat{\mathbf{g}}}_{{\varvec{v}}}\right)}^{2}$ are between 0.006 and 0.008

^**^Different letters in the superscripts denote statistically significant differences (P < 0.05)

^***^Subscripts unw, teqw, and tsw mean unweighted, equal weights across the traits, and unequal weights across the traits, respectively

Table 3 shows inflation biases of the multitrait predictions measured as the regression coefficients of the yield deviations on the GEBVs for the 1988 validation cows. The inflation bias was only significant for the SCC analysis without SNP weights, where there was a deflation bias. The analyses that used SNP weights yielded virtually unbiased genomic predictions.

**Table 3:** Inflation bias of genomic predictions measured as the regression coefficient of yield deviations on GEBVs for 1988 validation cows

	${b}_{{\text{y}}_{v},{\widehat{\text{g}}}_{v}}$^*^		
	Milk	Protein	SCC
SNP_unw_-BLUP^**^	1.08	1.07	1.19
SNP_teqw_-BLUP^**^	1.01	1.00	1.06
SNP_tsw_-BLUP^**^	0.99	0.98	1.01

^*^Standard errors of ${{\varvec{b}}}_{{\mathbf{y}}_{{\varvec{v}}},{\widehat{\mathbf{g}}}_{{\varvec{v}}}}$ are between 0.04 and 0.06

^**^Subscripts unw, teqw, and tsw mean unweighted, equal weights across the traits, and unequal weights across the traits, respectively

Alternative trait specific SNP weights were recently tested by [10] who used the marker specific scaling values of a previously conducted BayesA analysis [1] as weights. Likewise, [14] used the weights from a non-linear method introduced by [2] and compared them to the heuristic weights $2{p}_{j}\left(1-{p}_{j}\right){\widehat{b}}_{j}^{2}$ suggested by [15], where ${\widehat{b}}_{j}$ is the SNP effect from an unweighted SNPBLUP analysis. The $2{p}_{j}\left(1-{p}_{j}\right){\widehat{b}}_{j}^{2}$ weights yielded the highest increase of the prediction reliability of up to 12.7%, which is similar to our current results. However, the rational for the inclusion of the heterozygosity $2{p}_{j}\left(1-{p}_{j}\right)$ in the formula for the SNP weights is not clear, since variances of SNP effect sizes are not affected by the heterozygosity of the SNPs. However, the standard errors of the SNP effect estimates are approximately proportional to ${\left[2{p}_{j}\left(1-{p}_{j}\right)\right]}^{-\frac{1}{2}}$, which, when inserted into Eq. (1), implies that $2{p}_{j}\left(1-{p}_{j}\right){\widehat{b}}_{j}^{2}$ is approximately proportional to $L{R}_{tj}$ (although the SNP effect estimates in a GWAS and SNP-BLUP analysis differ). The rational of the GWABLUP SNP weights is that, in the BayesC model, the expected variance of a SNP effect is proportional to its posterior probability of having a non-zero effect.

It seems natural to combine the GWAS signals across the traits by a multitrait GWAS [6], which makes optimal use of the data. But multitrait GWAS analyses are computationally rather complicated, and simpler single-trait GWAS based approaches may be preferred. For instance, the results from single-trait GWAS analyses may be combined by a multitrait meta-GWAS analysis [16] which is based on summary statistics of the single-trait analyses. For SNP_tsw_-BLUP models, single-trait GWAS analyses may be directly used to provide the trait specific SNP weights. The accuracy of the resulting predictions will depend on the power of the single-trait SNP analyses. If the single-trait SNP analyses are not very powerful (do not result in clear genome-wide significant QTL signals), the use a multitrait GWAS analysis may be worthwhile.

Alternatively, a meta-analysis may be used to combine the GWAS signals across several data-sets [17] and thereby increase the power of the GWAS analysis. Although, GWAS analyses on older or other data where the LD patterns between SNPs and QTL may differ from the current data set, may result in less appropriate SNP weights. However, the most important SNPs with consistent LD across the data sets will still be upweighted. SNPs with inconsistent LD will obtain reduced effect estimates either because their high GWAS signal was combined with a low signal in the genomic prediction data or vice versa. Also, if several GWAS analyses have been applied on partial data sets, they can be combined by a meta-analysis GWAS, where weights of inconsistent SNPs (SNP estimates change sign between analyses) will be substantially reduced and may be artificially set to zero, since such SNPs are clearly not in consistent LD with the QTL. In addition, prior information on SNPs, such as whether they are synonymous mutations, can be implemented as SNP specific $\pi$-values, and thus affect $P{P}_{tj}$.

The trait specific SNP weights applied in Eq. (3) adjust the prior variances of the traits on a per SNP basis, but not the correlations between the traits. Equal correlations between the traits SNP effects were also suggested by [8, 10]. A more flexible model would estimate also prior correlations of SNP effects on a per (group of) SNP(s) basis. Gebreyesus et al. [18] estimated prior correlations for groups of SNPs and obtained improved prediction reliabilities for milk composition traits. More research will be needed to investigate whether SNP specific prior correlations would increase the reliabilities of the genomic predictions, or whether the standard errors of SNP specific correlations are too large for this approach to be beneficial.

There are two ways to increase the accuracy of genomic predictions: (i) to increase reference population size; and (ii) to (better) locate the causal variants and use this information in genomic prediction [19]. For large reference population sizes > 100,000 and prediction accuracies exceeding 90%, the scope for GWABLUP to further improve predictions is obviously limited. Multitrait GWABLUP concentrates on approach (ii), and may improve prediction reliabilities by up to 13% when reference population sizes are of limited size (tens of thousands), which may be due to small population sizes, no large-scale trait recording for (some of) the traits, or limited numbers of genotyped animals, or any combination of the above.

## Conclusions

A multitrait SNP-BLUP model was presented with trait specific SNP weights based on the GWABLUP approach. The model with trait specific SNP weights yielded EBVs with the highest reliability for all three traits analysed. For SCC, the model with identical SNP weights reduced the reliability of the EBV compared to unweighted SNP-BLUP, which was because the SNP weights were dominated by the milk production traits. This problem was remedied by the use of trait specific SNP weights. The multitrait GWABLUP models yielded up to 13% more reliable EBV compared to unweighted multitrait SNP-BLUP.

## Data Availability

Data are available upon request and approval of Geno SA.
